# Supplementary material for: Segmentally Duplicated Regulatory Elements Undergo Human-Specific Rewiring
Source: Mol Biol Evol. 2026 Jul 6;43(7):msag140. doi: 10.1093/molbev/msag140 (PMC13333887; doi:10.1093/molbev/msag140)
Supplement: msag140_Supplementary_Data [file msag140_supplementary_data.zip › suppTextFigs.pdf]

Supplementary Text and Figures

Seth Weaver, Craig B. Lowe
June 3, 2026

Related manuscript:
Weaver S., Lowe C.B. *Segmentally duplicated regulatory elements undergo human-specific rewiring.*

Supplementary Results

0.1 Maintenance of regulatory relationships

Many promoters maintain their regulatory activity following segmental duplication (Fraimovitch and Hagai, 2023). Maintenance of promoter activity following duplication can be grouped into two general categories with respect to the size of the duplication. The entire gene structure can be duplicated (i.e. promoter and all protein-coding exons) creating a duplicate copy of the original gene (Figure S4A). Alternatively, promoters can undergo smaller duplications where they are duplicated by themselves, or with only some of their accompanying exons, to become an alternative promoter of the same gene (Figure S4B), or a new gene based on a truncated version of the original gene (Figure S4C). We chose to focus on REF1 (Cluster 634) because it demonstrates both categories of promoter evolution.

The most straightforward case is when the entire gene structure is duplicated along with the promoter, likely resulting in a situation that is analogous to an upregulation of the original gene. REF1 is a family of 29 open elements where many are annotated as being promoters in the T2T gene set. While members of this REF are found on seven different chromosomes, 15 paralogs are located in the REPD and REPP SD clusters, which are 3.76 MB apart on chromosome 8 (Bosch et al., 2007) (Figure S5A). These SD clusters contain multiple gene families, including a *translation initiation factor IF-2-like* family. An example of a gene in this family is *LOC124906718* (Figure S4E), which underwent duplication via SD to create a human-specific copy, *LOC124906734* (Figure S4F) (Falker-Gieske, 2023). To verify that the promoters of these genes (which are members of REF1) are active, we targeted both with gRNAs in our CRISPRi screen. In cells that had gRNAs targeting REF1β (we use an additional letter to uniquely identify a member of a family), the downstream promoter of *LOC124906718*, we observed a 46% reduction in *LOC124906718* expression (Figure S4J) (L2FC = -0.89, raw $p < 10^{-5}$, BH FDR < 0.0001). Similarly, cell with gRNAs targeting REF1o, the downstream promoter of *LOC124906734*, had a 27% reduction in *LOC124906734* expression (Figure S4K) (L2FC = -0.46, raw $p < 10^{-4}$, BH FDR < 0.01). This duplication likely resulted in humans having greater expression of proteins with homology to *translation initiation factor IF-2-like* and this could potentially have effects on human-specific phenotypes since structural

variation affecting these clusters cause developmental delay (Barber et al., 2008; Yu et al., 2010). While there are a number of similar instances in the literature of promoters maintaining activity upon full gene duplication (Zhang et al., 2022; Soto et al., 2025), this confirms that we are able to detect such events with our CRISPRi screen in ESCs.

Along with promoters being duplicated with their entire gene structure, they can also be duplicated in smaller tandem events where they continue to act on the original gene as an alternative promoter, or create a new gene based on a truncation of the original gene structure. Interestingly, the *translation initiation factor IF-2-like* genes from the first example not only have one REF1 element acting as a promoter, but have two REF1 elements, each acting as alternative promoters. Both overlap regions of open chromatin and serve as transcription start sites, based on 5' RNA-seq alignments (Figure S4E,F). These alternative promoters are the result of an ancient tandem duplication event that formed a common duplicon and was subsequently spread around the genome through further SDs. While we are uncertain of the original tandem duplication event, we observed a human-specific tandem duplication on chromosome 3, creating the dual promoter configuration for a gene in humans, while the other great apes appear to have a single promoter driving expression of *LOC128966749* (Figure S4G). Using CRISPRi, we targeted REF1b, the downstream promoter of *LOC128966749*, and observed a 60% reduction in *LOC128966749* expression (Figure S4L) (L2FC = -1.31, raw $p < 10^{-20}$, BH FDR < 0.0001). These examples show how tandem duplications of promoters can create novel isoforms and SDs of entire genes can expand gene families throughout the genome.

Along with tandem duplications creating alternative promoters, partial duplications can be inserted far from their source copy. Partial gene duplications have been shown to remain functional, and even create fusion transcripts (Dougherty et al., 2018). Therefore, we validated the REF1 promoter elements in two truncated *translation initiation factor IF-2-like* duplications that have created noncoding RNAs. *OR7E160P* is a gene duplication of the first two exons of the previously targeted *translation initiation factor IF-2-like* protein-coding genes, located at one end of the REPD cluster (Figure S4H). We used 5'-capture RNA-seq data to refine the gene annotation at the locus and predict that *OR7E160P* has the common dual-promoter structure previously observed with other REF1 genes (Figure S4H). When the downstream element in the tandem pair is targeted, REF1f, we observed a 37% reduction in OR7E160P expression (Figure S4M) (L2FC = -0.66, raw $p < 10^{-6}$, BH FDR < 0.0001). There is an additional noncoding RNA gene with a REF1 promoter outside the REPP and REPD SD clusters, *LOC124906423*, within an SD on chromosome 4 (Figure S4I). Similar to other genes with REF1 promoters, *LOC124906423* expression was repressed by 52% in cells with promoter targeting gRNAs (Figure S4N) (L2FC = -1.07, raw p. = 0.0014, BH FDR < 0.1). We therefore suggest that REF1 elements have remained functional promoters even upon ancient or partial gene-duplication events. While there are number of similar examples of partially duplicated genes maintaining promoter activity following a segmental duplication (Fiddes et al., 2018), the repression of five genes with REF1 promoters in response to a common CRISPRi gRNA stimulus shows the maintenance of promoter activity in ESCs after duplication (Figure S4D), and

indicates that our multi-loci gRNA targeting framework can concurrently assess the *in vitro* function of multiple sites in the genome.

References

References

Barber, J. C., Maloney, V. K., Huang, S., Bunyan, D. J., Cresswell, L., Kinning, E., Benson, A., Cheetham, T., Wyllie, J., Lynch, S. A., *et al.*, 2008. 8p23. 1 duplication syndrome; a novel genomic condition with unexpected complexity revealed by array cgh. *European journal of human genetics*, **16**(1):18–27.

Bosch, N., Cáceres, M., Cardone, M. F., Carreras, A., Ballana, E., Rocchi, M., Armengol, L., and Estivill, X., 2007. Characterization and evolution of the novel gene family fam90a in primates originated by multiple duplication and rearrangement events. *Human molecular genetics*, **16**(21):2572–2582.

Dougherty, M. L., Underwood, J. G., Nelson, B. J., Tseng, E., Munson, K. M., Penn, O., Nowakowski, T. J., Pollen, A. A., and Eichler, E. E., 2018. Transcriptional fates of human-specific segmental duplications in brain. *Genome research*, **28**(10):1566–1576.

Falker-Gieske, C., 2023. Transcriptome driven discovery of novel candidate genes for human neurological disorders in the telomer-to-telomer genome assembly era. *Human Genomics*, **17**(1):94.

Fiddes, I. T., Lodewijk, G. A., Mooring, M., Bosworth, C. M., Ewing, A. D., Mantalas, G. L., Novak, A. M., van den Bout, A., Bishara, A., Rosenkrantz, J. L., *et al.*, 2018. Human-specific notch2nl genes affect notch signaling and cortical neurogenesis. *Cell*, **173**(6):1356–1369.

Fraimovitch, E. and Hagai, T., 2023. Promoter evolution of mammalian gene duplicates. *BMC biology*, **21**(1):80.

Soto, D. C., Uribe-Salazar, J. M., Kaya, G., Valdarrago, R., Sekar, A., Haghani, N. K., Hino, K., La, G., Mariano, N. A. F., Ingamells, C., *et al.*, 2025. Human-specific gene expansions contribute to brain evolution. *Cell*, **188**(19):5363–5383.

Yu, S., Fiedler, S., Stegner, A., and Graf, W. D., 2010. Genomic profile of copy number variants on the short arm of human chromosome 8. *European journal of human genetics*, **18**(10):1114–1120.

Zhang, D., Leng, L., Chen, C., Huang, J., Zhang, Y., Yuan, H., Ma, C., Chen, H., and Zhang, Y. E., 2022. Dosage sensitivity and exon shuffling shape the landscape of polymorphic duplicates in drosophila and humans. *Nature Ecology & Evolution*, **6**(3):273–287.

Supplementary Figures

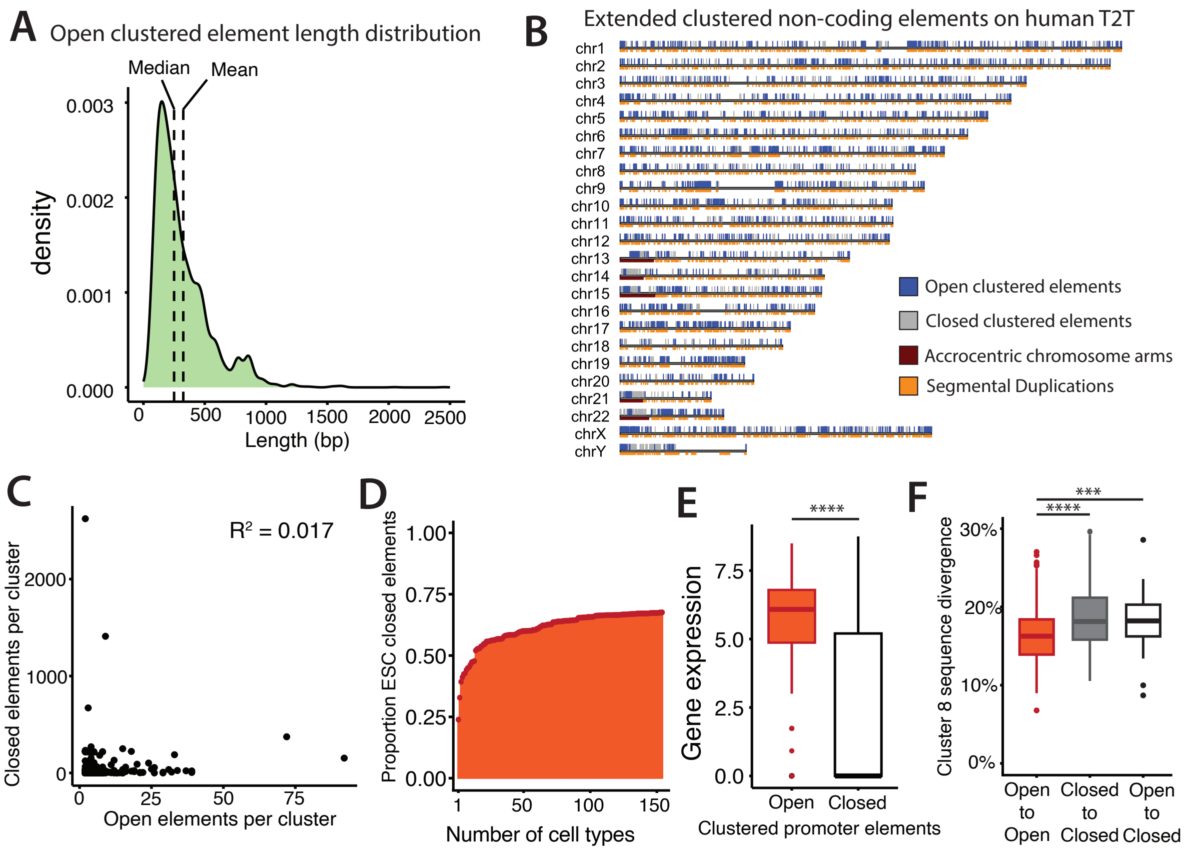

Figure S1: **Open and closed clustered elements harbor differences in ESCs.** **(A)** Size distributions of open ESC clustered elements. Eleven elements larger than 2500 bp were filtered out for ease of visualization. **(B)** Location of embryonic stem cell (ESC) open clustered elements, closed clustered elements, segmental duplications and acrocentric chromosome arms in the human T2T reference genome. **(C)** Correlation of number of open and closed ESC elements per cluster. **(D)** Proportion of ESC closed elements that have accessible chromatin in other ENCODE cell types as increasing cell types are considered. **(E)** Comparison of gene expression between gene with open vs closed ESC promoters. Y-axis values reflect Log10-transformed fragments per kilobase plus a pseudocount. (Wilcoxon; ****: $p < 0.0001$). **(F)** Percent divergence between extended Cluster 8 elements, comparing sequence divergence between open elements, between open and closed elements, and between closed elements. (t-test; ***: $p < 0.001$, ****: $p < 0.0001$)

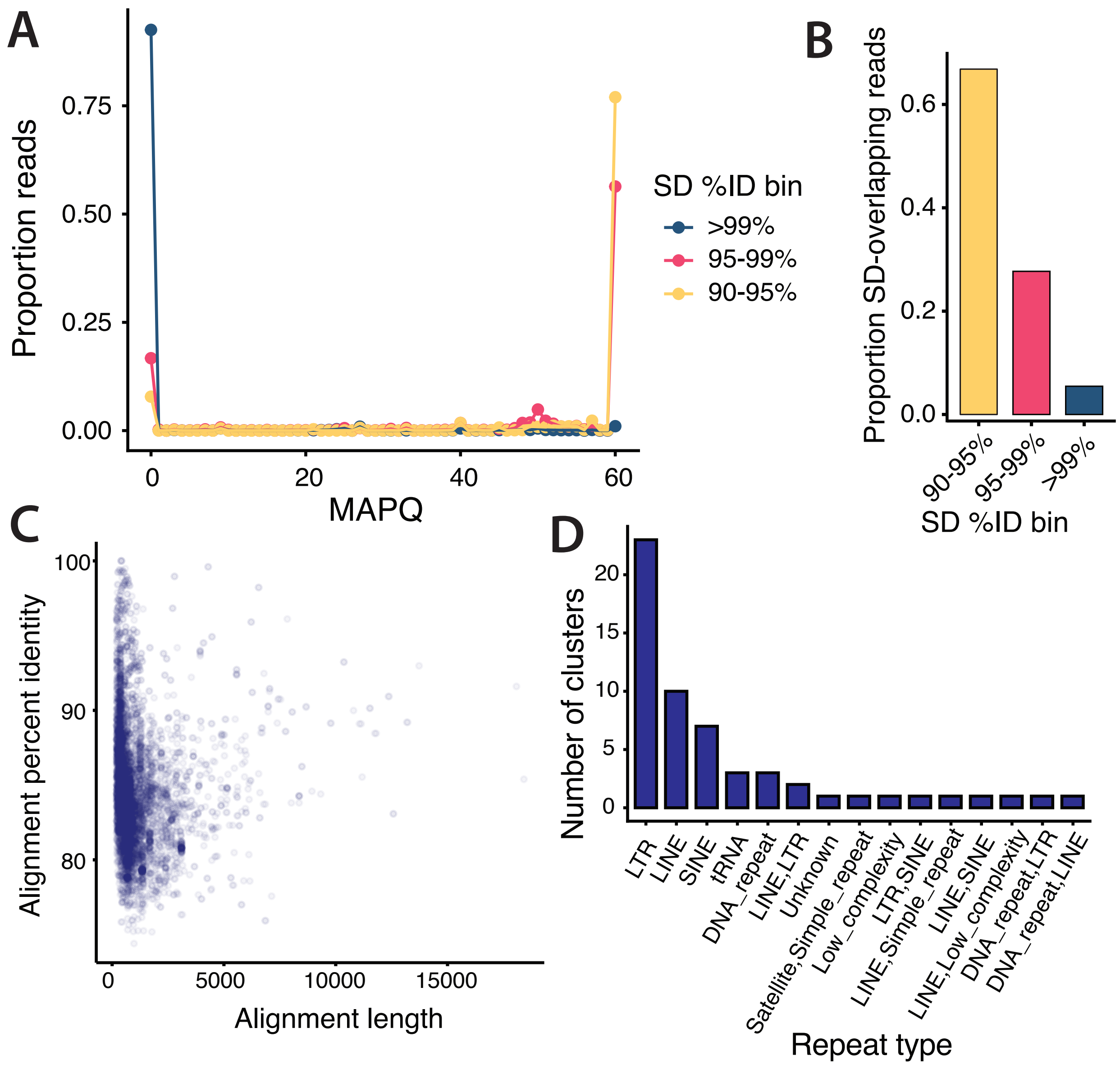

Figure S2: **Additional genomic mechanisms of ESC cluster evolution.** **(A)** Proportion of ESC ATAC-seq reads at each mapping quality score (MAPQ) that overlap three distinct bins of SD percent identity. **(B)** Proportion of total SD-overlapping reads that are contained within the three bins of SD percent identity. **(C)** Alignment length and percent identity statistics for self-alignments overlapping non-SD clusters. **(D)** Number of ESC clusters overlapping each mobile element class.

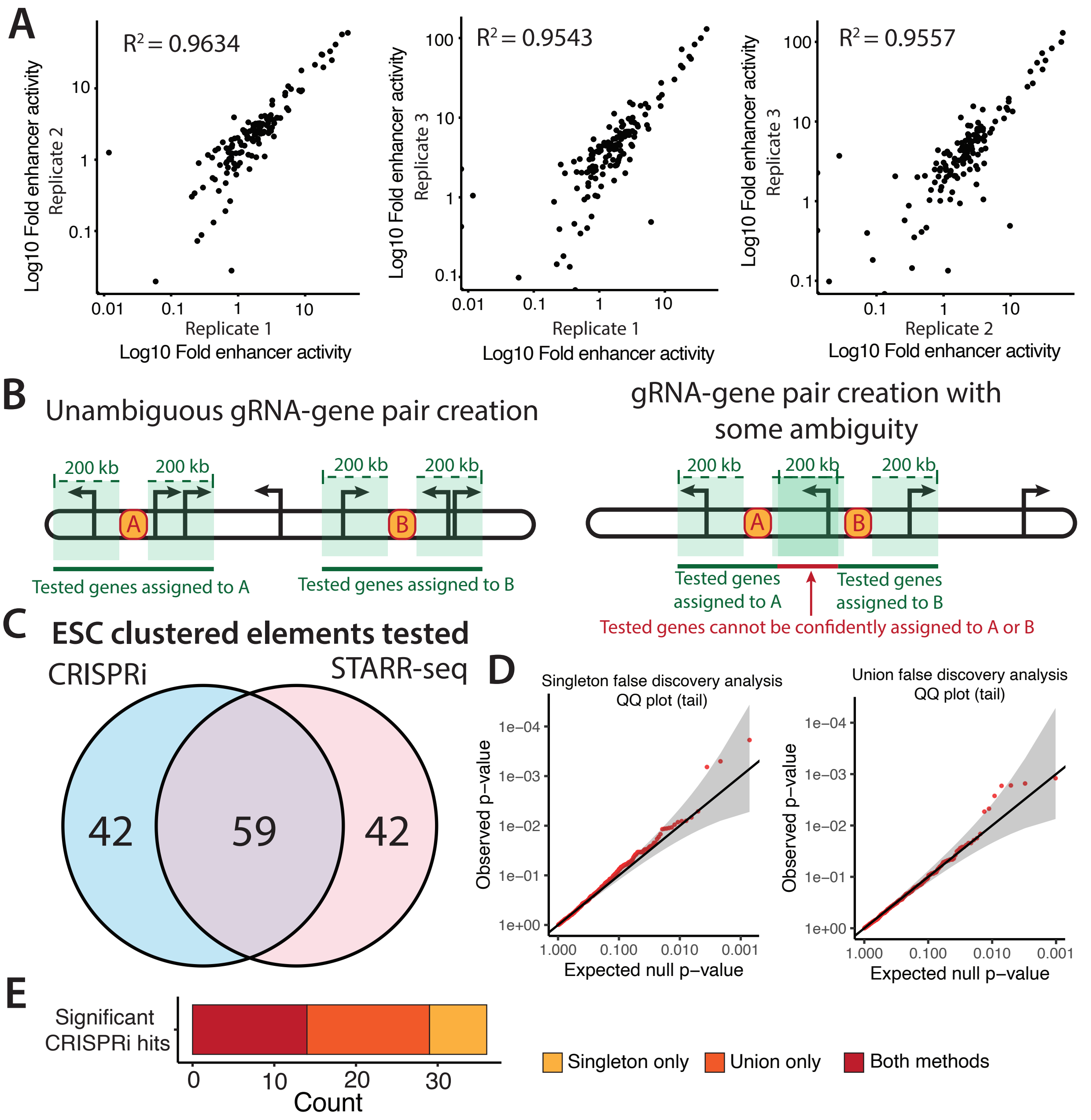

Figure S3: **Functional data analysis quality (A)** Correlation of three STARR-seq replicates performed in H9 ESCs. Axes are Log10 transformed. **(B)** Representation of gRNA-gene pair creation for association testing by sceptre with a 200kb distance filter, where elements "A" and "B" are paralogous in a cluster. Arrows denote location of genes in the model. **(C)** The number of ESC clustered elements that were tested using either STARR-seq, CRISPRi, or with both assays. **(D)** Quality-control false discovery QQ plots produced by sceptre for both singleton and union analyses. No false positives were detected in either analysis. **(E)** Number of significant clustered element CRISPRi hits that were found in the singleton analysis, the union analysis or both analysis methods.

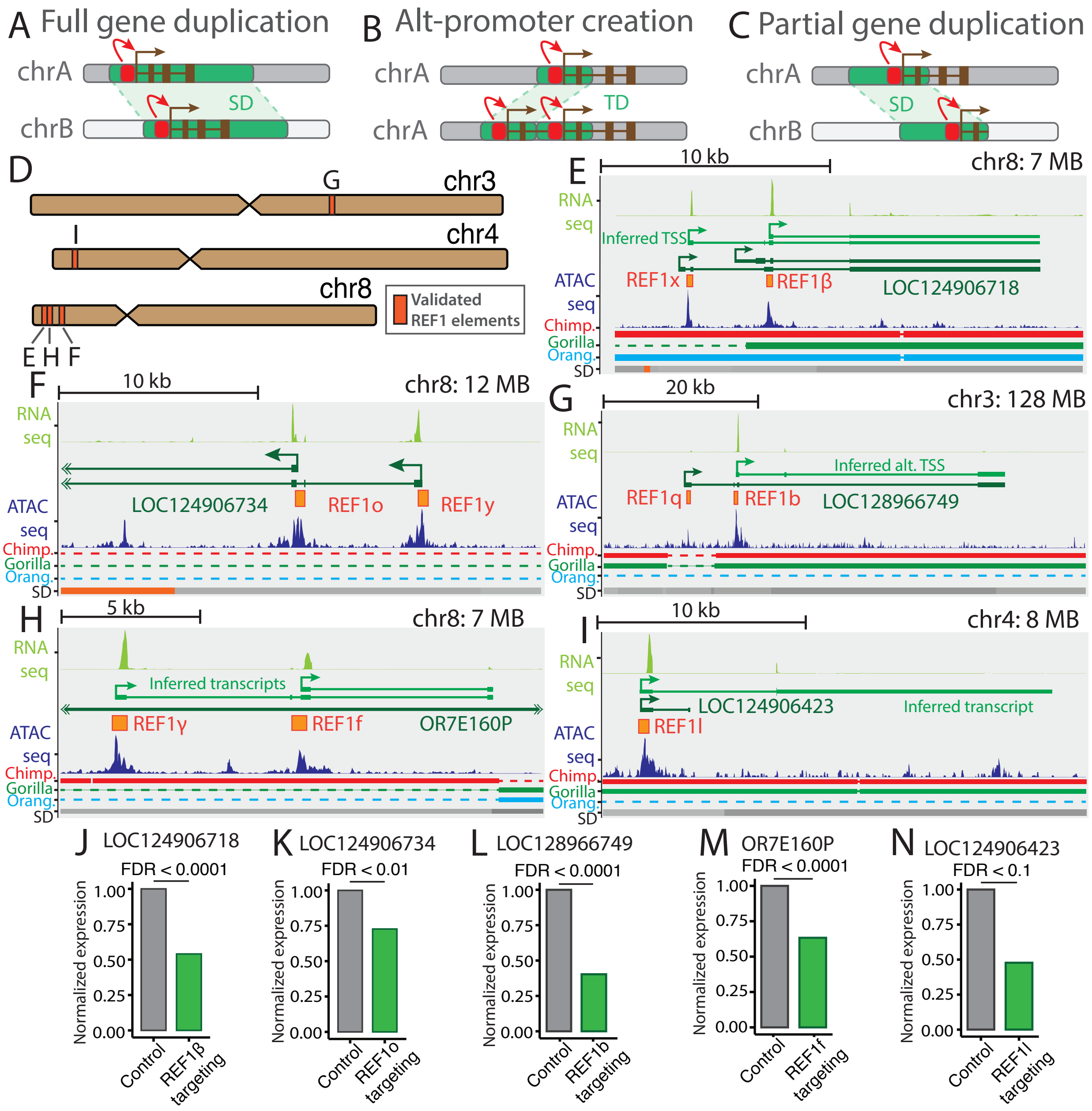

Figure S4: **REF1 proximally regulates genes.** **(A-C)** Models of complete or partial gene duplication. The number of times we observed each scenario for functionally tested REF1 elements is as follows: A = 1; B = 1; C = 4. **(D)** Schematic of validated REF1 promoters, where letters correspond to figure panels showing additional genomic context. **(E-I)** Genomic contexts of REF1 promoter elements with significant CRISPRi signal. We used our 5'-capture RNA-seq from ESCs to refine the gene models near REF1 promoter genes, particularly with respect to transcription start site. To improve visualization, we show the inferred transcripts based on the ESC RNA-seq alignments, as well as the ESC RNA-seq alignments. Sequence alignments to T2T primate genomes are shown, with horizontal bars showing homology, and dashed lines showing alignment gaps (Chimp. = Chimpanzee; Orang. = Orangutan) **(J-N)** Normalized gene expression upon CRISPRi repression of REF1 elements (Benjamini-Hochberg FDR < 0.1).

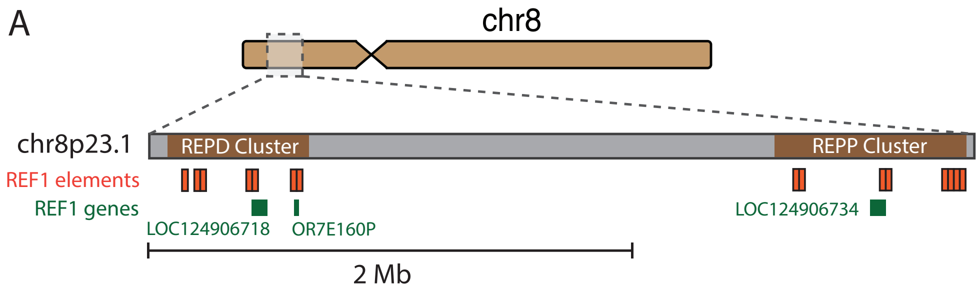

Figure S5: **Chromosome 8 SD clusters harbor REF1 promoters (A)** Graphical representation of REF1 elements within segmental duplication clusters on chromosome 8p23.1.

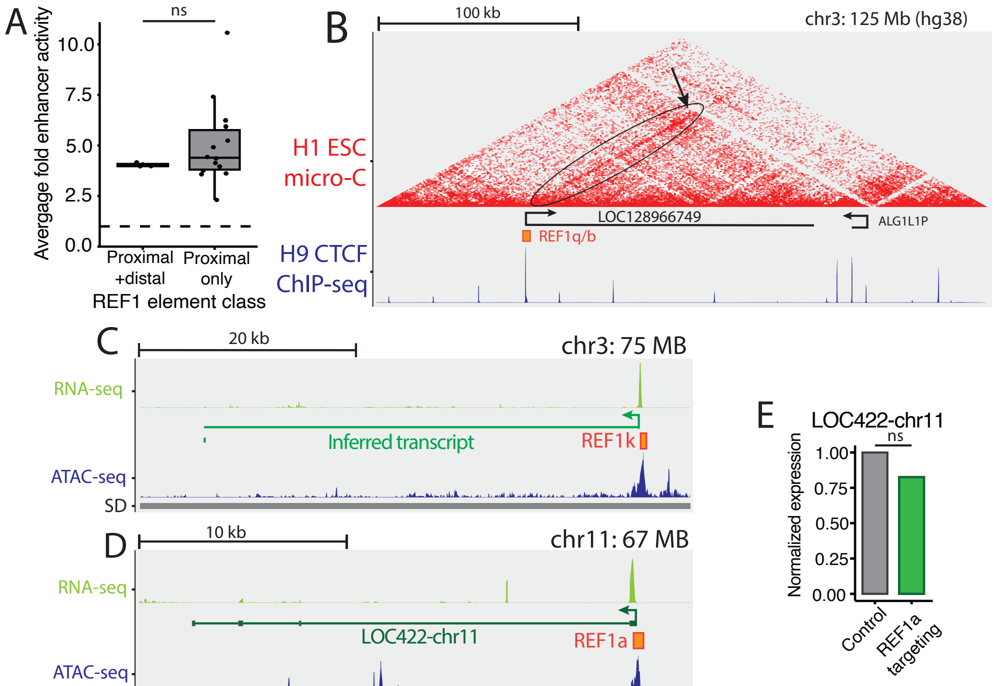

Figure S6: **REF1 associated data (A)** Comparison of enhancer activity between CRISPRi-targeted REF1 elements that had proximal and distal regulatory activity compared to REF1 elements that had proximal activity only, measured by STARR-seq (Wilcoxon test, ns: $p > 0.05$). **(B)** 3D genome organization of the *LOC128966749-ALG1L1P* promoter-promoter interaction visualized on the hg38 genome assembly. The oval shows the stripe domain facilitated by the *LOC128966749* promoter, and the arrow shows the interaction with the *ALG1L1P* promoter. **(C)** Genomic context for REF1k. A transcription start site was inferred based on 5'-capture RNA-seq from ESCs and a gene model lifted from the hg38 assembly. **(D)** Genomic context for REF1a. *LOC422-chr11* was lifted from the hg38 reference. **(E)** Normalized *LOC422-chr11* expression upon repression of REF1a with CRISPRi (ns: Benjamini-Hochberg FDR > 0.1)

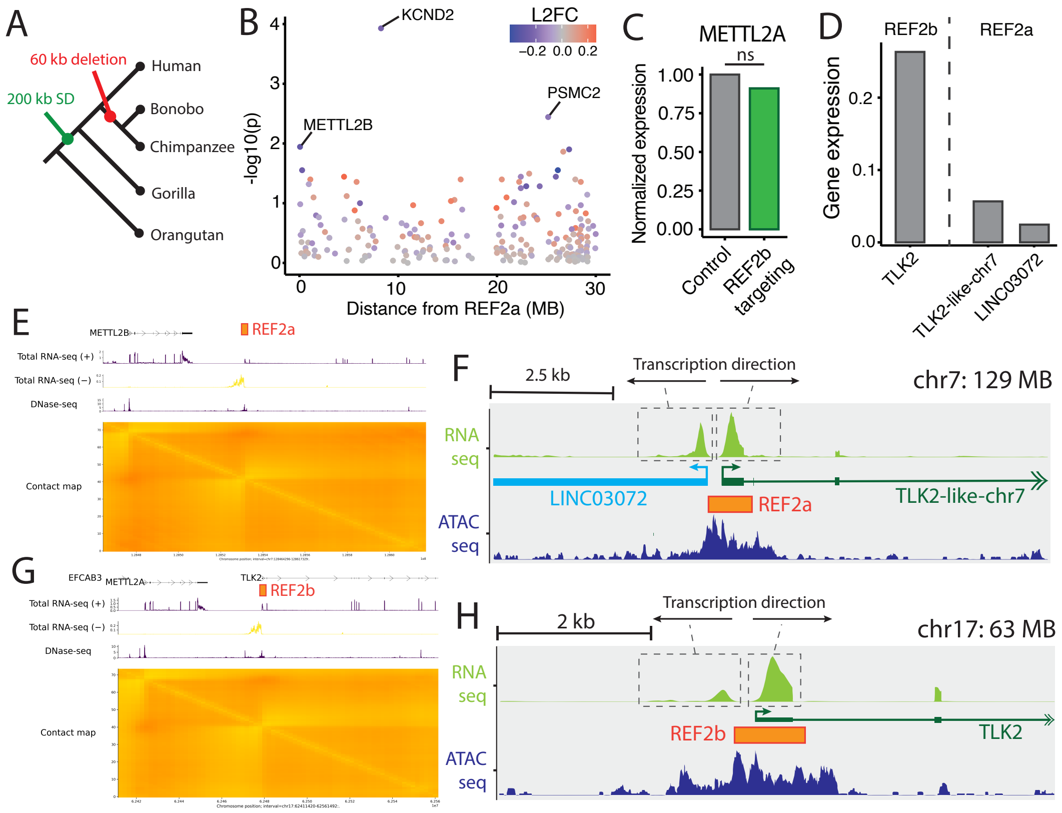

Figure S7: **REF2 associated data (A)** Primate phylogeny with arbitrary branch lengths showing the duplication history of the segmental duplication that created REF2a. **(B)** All gene expression responses within 30MB on either side of REF2a following CRISPRi repression. **(C)** Normalized *METTL2A* expression upon repression of REF2b with CRISPRi (ns: Benjamini-Hochberg FDR > 0.1). **(D)** Average read counts per cell of genes with REF2 promoters, as calculated by Seurat. Cells that recieved a REF2-targeting guide were filtered out. The REF2 element that act as the promoter are shown above their respective genes. **(E,G)** Genomic context of REF2a and REF2b showing predicted transcription, chromatin accessibility, and 3D contacts. **(F,H)** Genomic context of REF2a and REF2b showing divergent transcription in ESCs. The genes proximal to REF2a, *TLK2-like-chr7* and *LINC03072*, are lifted onto the human T2T assembly from hg38.

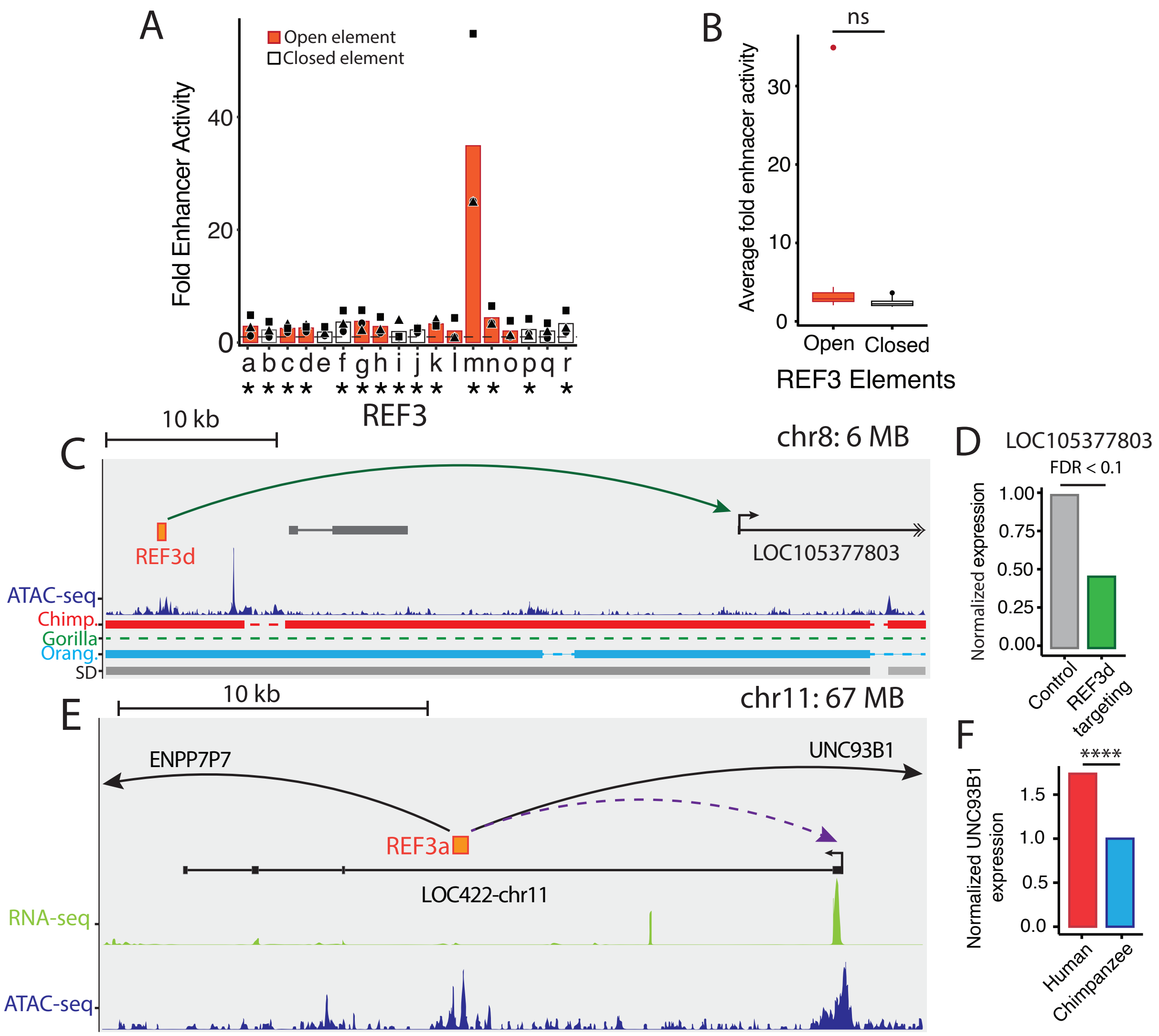

Figure S8: **REF3 rewiring associated data (A)** Fold enhancer activity of REF3 elements over negative controls. Data point shape denotes replicate number. Dotted line is the average of negative controls (Stauffer's method, *: $p < 0.01$). **(B)** Comparison of fold enhancer activity in REF3 between open and closed elements (Wilcoxon; ns: $p > 0.5$). **(C)** Genomic context for REF3d. Curved arrows represent a distal regulatory connection. **(D)** Normalized *LOC105377803* expression upon CRISPRi repression of REF3a (Benjamini-Hochberg FDR > 0.1) **(E)** Genomic context of REF3a. The *LOC422-chr11* gene model was lifted onto human T2T from hg38. Curved arrows represent distal regulatory interactions. **(F)** Human *UNC93B1* expression normalized to chimpanzee *UNC93B1* expression. Data from *Gokhman et. al., 2021*, using human-chimpanzee allotetraploid cells (Differential expression test; $p < 0.0001$: ****).

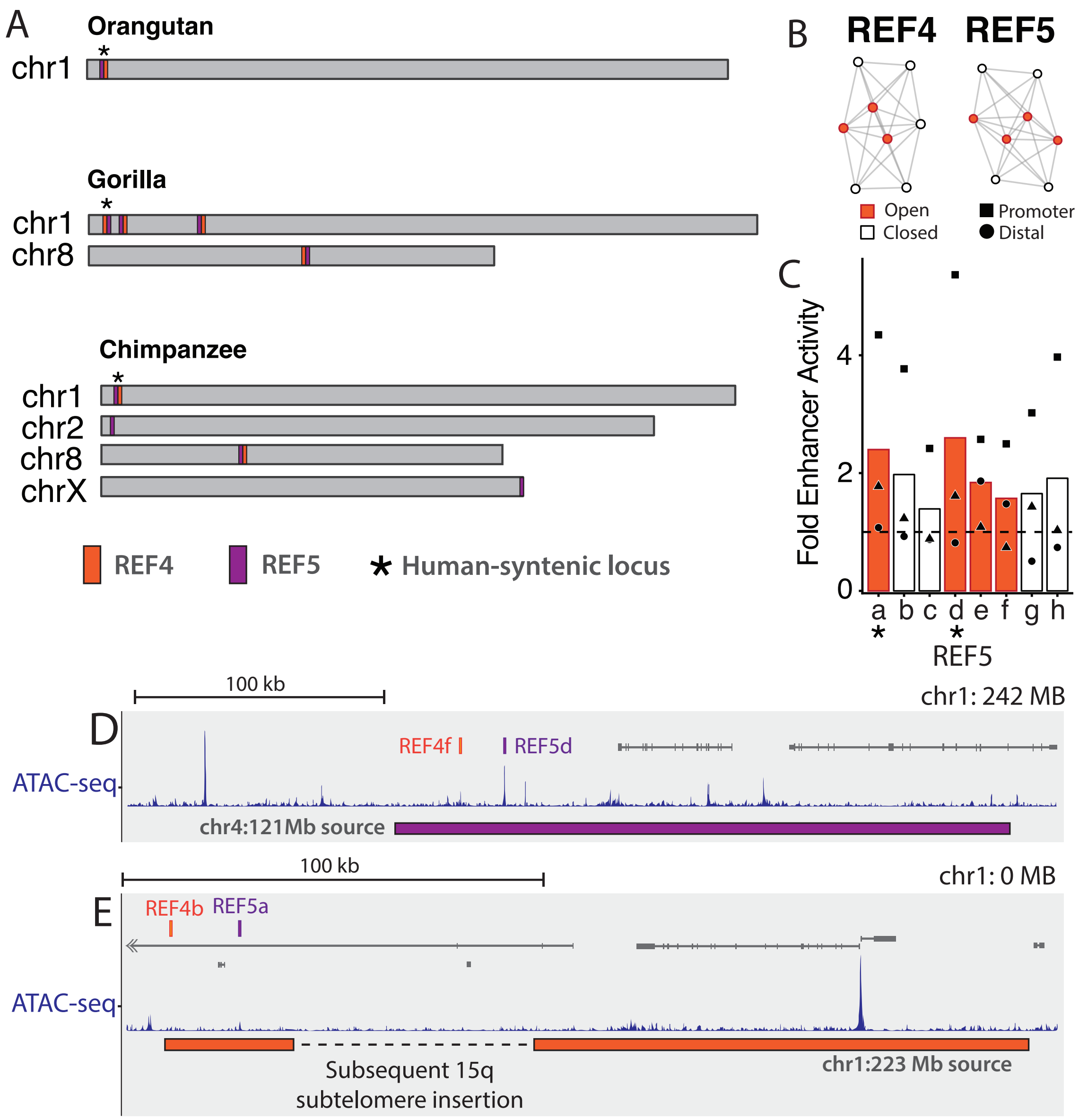

Figure S9: **Lineage-specific duplications of REF4 and REF5.** **(A)** Locations of REF4 and REF5 elements in T2T primate genomes. **(B)** REF4 and REF5 visualized with edge lengths corresponding to percent divergence between noncoding element nodes. **(C)** Fold enhancer activity of REF5 elements over negative controls, measured by STARR-seq. Data point shape denotes replicate number. Dotted line is the average of negative controls (Stauffer's method, *: $p < 0.01$). **(D-E)** Genomic context showing the source sequence for human-specific SDs containing REF4/5 elements.

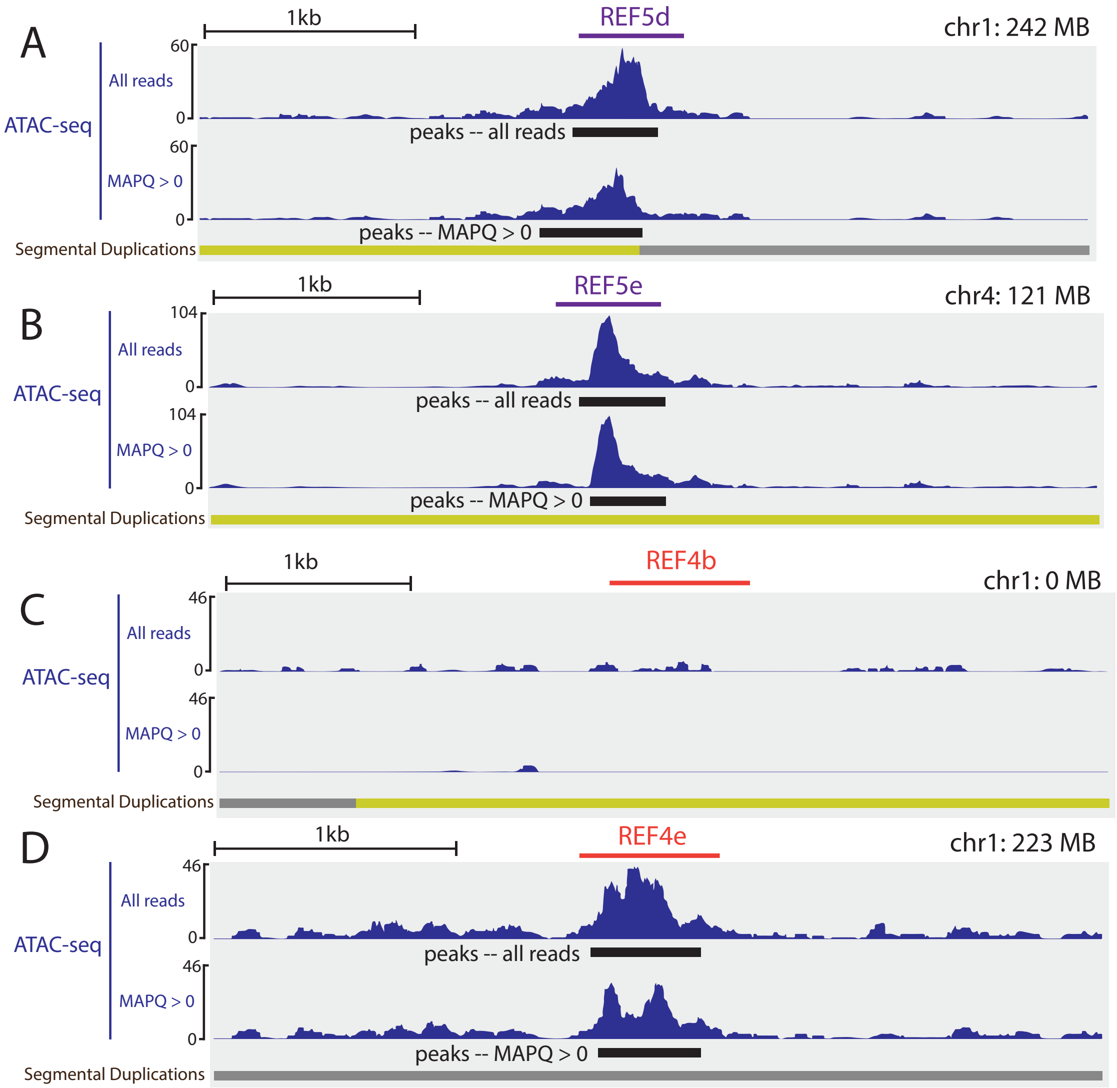

Figure S10: **REF4 and REF5 elements have robust open chromatin signal. (A-D)** Genomic context of REF4 and REF5 elements targeted with CRISPRi guides. Chromatin accessibility (ATAC-seq) data is shown representing all reads and the subset of reads with mapping quality scores (MAPQ) > 0. Open chromatin peaks were called on both ATAC-seq subsets and shown below their respective coverage tracks.
